# Langzeitverlauf von Effizienz und Sicherheit der CD19-CAR T-Zell-Therapie beim systemischen Lupus erythematodes

**DOI:** 10.1007/s00393-025-01705-0

**Published:** 2025-09-04

**Authors:** Jule Taubmann, Sebastian Böltz, Melanie Hagen, Andreas Wirsching, Fabian Müller, Simon Völkl, Soraya Kharboutli, Silvia Spörl, Panagiotis Garantziotis, Michael Aigner, Ricardo Grieshaber-Bouyer, Andreas Mackensen, Georg Schett

**Affiliations:** 1 Medizinische Klinik 3 – Rheumatologie und Immunologie, Friedrich-Alexander-Universität Erlangen-Nürnberg und Universitätsklinikum Erlangen, Ulmenweg 18, 91054 Erlangen, Deutschland; 2 Deutsches Zentrum für Immuntherapie (DZI), Friedrich-Alexander-Universität Erlangen-Nürnberg und Universitätsklinikum Erlangen, Erlangen, Deutschland; 3 Medizinische Klinik 5 – Hämatologie und Onkologie, Friedrich-Alexander-Universität Erlangen Nürnberg und Universitätsklinikum Erlangen, Erlangen, Deutschland

**Keywords:** B‑Zellen, Zelluläre Therapie, Remission, Chimäre Antigenrezeptor-T-Zellen, Sicherheitsprofil, B‑cells, Cellular therapy, Remission, Chimeric antigen receptor T‑cells, Safety profile

## Abstract

**Hintergrund:**

Chimäre Antigenrezeptor(CAR)-T-Zellen haben sich als effektive Therapieoption in der Behandlung von B‑Zell-assoziierten Malignomen etabliert. Neben malignen B‑Zellen können auch autoreaktive B‑Zellen ein Ziel der anti-CD19-gerichteten CAR-T-Zellen (CD19-CAR-T-Zellen) darstellen. B‑Zellen sind beim systemischen Lupus erythematodes (SLE) maßgeblich an der Produktion pathogener Autoantikörper beteiligt und fördern sowohl den Krankheitsausbruch als auch das Fortschreiten der Erkrankung. In unseren bisherigen Forschungsarbeiten als auch in weiteren Fallanalysen konnte gezeigt werden, dass der Einsatz der CD19-CAR-T-Zell-Therapie bei schwerem, therapierefraktärem Verlauf des SLE eine vielversprechende Wirksamkeit sowie ein akzeptables Sicherheitsprofil aufweist.

**Ziel der Arbeit:**

Es wird ein Fazit zum aktuellen Stand bezüglich Wirksamkeit und Sicherheit der CD19-CAR-T-Zell-Therapie beim systemischen Lupus erythematodes (SLE) gegeben.

**Material und Methode:**

PatientInnen mit progredientem, therapierefraktärem SLE erhielten im Rahmen eines individuellen Heilversuches eine autologe CD19-CAR-T-Zell-Therapie (MB19,1, Miltenyi Biotec, Bergisch Gladbach, Deutschland) und werden regelmäßig an unserem Zentrum nachbeobachtet.

**Ergebnisse:**

Elf PatientInnen mit progredientem, therapierefraktärem SLE erhielten im Rahmen eines individuellen Heilversuches eine autologe CD19-CAR-T-Zell-Therapie. Die mediane Nachbeobachtungszeit beträgt 2,5 Jahre (0,5 bis 4 Jahre). Alle PatientInnen erreichten innerhalb von 6 Monaten eine DORIS-Remission (Definition of Remission in SLE). Die immunsuppressive Therapie wurde bei allen PatientInnen vollständig abgesetzt. Fünf der 11 PatientInnen hatten ein „cytokine release syndrome“ (CRS) ersten Grades. CRS Grad 2 wurde nur 1‑malig beobachtet. Höhergradige CRS sind in dieser Kohorte nicht aufgetreten. Bei unseren SLE-PatientInnen war keine Neurotoxizität („immune effector cell-associated neurotoxicity syndrome“ [ICANS]) festzustellen. Alle PatientInnen befinden sich aktuell in einer anhaltenden und medikamentenfreien Remission. Wir verzeichneten bei einer Patientin einen therapiebedürftigen SLE-Schub. Erste Daten deuten trotz ähnlicher CD19-CAR-T-Zell-Expansion und Kinetik auf ein besseres Sicherheitsprofil der CD19-CAR-T-Zell-Therapie bei SLE im Vergleich zu Lymphomkohorten hin. Bei SLE-Patientinnen stellt sich die adaptive Immunität nach einer CD19-CAR-T-Zell-Therapie zudem schnell wieder her.

**Schlussfolgerungen:**

Der Einsatz von CD19-CAR-T-Zellen bei PatientInnen mit schwerem Verlauf des SLE erweist sich als sicher und wirksam.

## Abstract (en)

**Background:**

Chimeric antigen receptor T-cells (CAR T-cells) are an effective therapeutic approach in the treatment of B-cell driven malignancies. In addition to malignant B-cells, autoreactive B-cells are important targets for CD19 CAR T-cells, as they are a source of autoantibody production and support both the onset and progression of systemic lupus erythematosus (SLE). We and others have shown that their use in severe and therapy-refractory cases of SLE is effective and, moreover, safe.

**Objective:**

The current status and an interim analysis of the efficacy and safety of CD19-CAR T‑cell therapy in SLE.

**Material and methods:**

Patients with severe, treatment-refractory, and active SLE received autologous CD19-CAR T‑cell therapy (MB19.1, Miltenyi Biotec, Bergisch Gladbach, Germany) as part of an individual compassionate treatment attempt and are regularly followed up at our center.

**Results:**

11 patients with progressive, therapy-refractory SLE received an autologous CD19 CAR T-cell therapy as part of an individual treatment attempt. The median follow-up time is 2.5 years [0.5 – 4 years]. All patients achieved a DORIS remission within 6 months. Immunosuppressive therapy was completely discontinued in all patients. Five out of the 11 patients experienced grade 1 cytokine release syndrome (CRS). Grade 2 CRS was observed only once. No higher-grade CRS occurred in this cohort. So far, no neurotoxicity (immune effector cell-associated neurotoxicity syndrome, ICANS) has been observed in our SLE patients. All patients remain in a sustained and drug-free remission to date. One patient experienced an SLE flare. Initial data, despite similar CD19 CAR T-cell expansion and kinetics, suggest a better safety profile of CD19 CAR T-cell therapy in SLE compared to lymphoma cohorts. Additionally, adaptive immunity in SLE patients recovers rapidly after CD19 CAR T-cell therapy.

**Conclusion:**

The use of CD19-CAR T‑cells in patients with severe SLE proved to be safe and effective.

Systemischer Lupus erythematodes (SLE) ist eine Autoimmunerkrankung, die sich durch eine B‑Zell-getriebene Produktion von Autoantikörpern gegen doppelsträngige Desoxyribonukleinsäure (dsDNA) und Kernproteine wie Nukleosomen oder Histonen kennzeichnet. Diese Autoantikörper können zu Immunkomplexen ausfallen und zu Entzündungen in verschiedenen Organen führen [1]. Die Inzidenzrate des SLE liegt bei etwa 6 bis 7 pro 100.000 Personen und betrifft v. a. junge Frauen im gebärfähigen Alter [2]. Trotz intensiver immunsuppressiver Therapie leiden einige SLE-PatientInnen an schweren und behandlungsresistenten Erkrankungsverläufen, die mit einem hohen Risiko für Organschäden und erhöhter Mortalität einhergehen. Außerdem gehen lebenslange immunsuppressive Therapien ebenfalls mit teils schwerwiegenden Komplikationen einher.

Die B‑Zell-vermittelte Produktion von Autoantikörpern wird als wesentlicher Schritt in der SLE-Immunpathologie gesehen [3]. Die B‑Zell gerichtete Therapie bei SLE war lange Zeit auf Antikörper beschränkt, die entweder B‑Zellen abbauen (z. B. durch Bindung des B‑Zell-spezifischen Oberflächenmoleküls CD20) [4] oder ihre Aktivierung und Reifung hemmen (z. B. durch Bindung des B‑Zell-aktivierenden Faktors [BAFF]) [5]. Diese Behandlungen zielen in der Regel darauf ab, die Entzündung zu kontrollieren, greifen dabei aber nicht tief in die Autoimmunpathologie ein und zeigten teilweise eine fehlende Gewebsinfiltrationstiefe [6]. Um diese Lücken zu schließen und eine kurzweilige, aber effektive B‑Zell-Depletion auch im Gewebe zu erreichen, haben wir auf eine zelluläre Technologie aus der Hämatoonkologie zurückgegriffen. Innerhalb weniger Jahre hat die sog. anti-CD19-gerichtete chimäre Antigenrezeptor(CAR)-T-Zell-Therapie die Behandlung von B‑Zell-assoziierten Malignomen revolutioniert [7, 8]. Neben malignen B‑Zellen stellen auch autoreaktive B‑Zellen ein prinzipielles Ziel der CD19-CAR-T-Zellen dar. Wir und andere haben den erfolgreichen Einsatz bei Autoimmunerkrankungen wie SLE, idiopathischer inflammatorischer Myositis (IIM) und diffus kutaner systemischer Sklerose (SSc) gezeigt [9–14]. Zudem konnte eine vollständige Depletion von B‑Zellen auch in sekundären lymphatischen Geweben von SLE-PatientInnen nach einer CD19-CAR-T-Zell-Therapie erreicht werden [15].

## Material und Methoden

### Anti-CD19-CAR-T-Zell-Therapie

PatientInnen mit schwer verlaufendem, therapierefraktärem und aktivem SLE erhielten im Rahmen eines individuellen Heilversuches eine autologe CD19-CAR-T-Zell-Therapie. Hierbei zeigte sich eine schwere organbedrohliche Krankheitsaktivität, die auf Standardtherapien nicht oder nur unzureichend angesprochen hat (Tab. 1). Die CD19-CAR-T-Zellen MB-CART19,1 (Miltenyi Biotec, Bergisch Gladbach, Deutschland) wurden, wie zuvor beschrieben, hergestellt [16–18]. Alle SLE-Behandlungen wurden vor der Verabreichung der CAR-T-Zellen gestoppt. Nach der Standardlymphozytendepletion mit Cyclophosphamid (1 × 1000 mg/m^2^) und Fludarabin (3-mal 25 mg/m^2^) wurde eine einzelne Infusion von 1‑mal 10^6^ CAR-T-Zellen/kg Körpergewicht verabreicht. In den ersten 10 Tagen nach der Verabreichung von CAR-T-Zellen wurden die PatientInnen stationär nachbeobachtet, dann wöchentlich bis zum Ende des ersten Monats, dann monatlich für 3 Monate und danach alle 3 Monate bis zu alle 6 Monate.

**Tab. 1:** Demografische Ausgangsdaten von 11 SLE(systemischer Lupus erythematodes)-PatientInnen, die mit einer CD19-CAR(chimärer Antigenrezeptor)-T-Zell-Therapie behandelt wurden

Patient	1	2	3	4	5	6	7	8	9	10	11
Alter (J.)	20	22	22	24	18	38	33	35	24	20	41
Geschlecht (w/m)	F	M	F	F	F	F	F	F	F	M	F
Erkrankungsdauer (J.)	4	1	6	8	3	18	1	20	2	1	2
Anzahl (Vor-)Therapien	6	5	4	8	5	14	7	13	7	6	5
Baseline SLEDAI (Punktzahl)	16	16	10	8	9	16	10	22	18	18	18
Baseline C3 (mg/dl) (Norm 81–157 mg/dl)	49	43	56	88	68	33	49	25	70	96	86
Dauer B-Zell-Aplasie (Tage)	148	196	120	93	63	205	58	225	172	199	n. d.
Lupusnephritis	+	+	+	+	+	+	+	+	+	–	–
ZNS-Beteiligung, Myelitis	–	–	–	–	–	–	–	–	+	+	+
Follow-up (Jahre)	4	3,5	3,5	3	3	3	2,5	2	1,5	1	0,5
Serokonversion	+	+	+	+	+	+	+	+	–	+	+
SLEDAI (letzte Visite)	0	0	0	0	0	0	0	0	22	0	0
DORIS-Remission (letzte Visite)	+	+	+	+	+	+	+	+	–	+	+
CRS (Grad 0–4)	0	1	1	1	0	1	0	1	2	0	0
ICANS (Grad 0–4)	0	0	0	0	0	0	0	0	0	0	0
ICAHT (frühe Neutropenie)	1	1	1	0	1	1	1	1	0	1	1
ICAHT (späte oder aplastische Neutropenie)	0	0	0	0	0	0	0	0	0	0	0
Schwere Infektionen	0	0	0	0	0	0	0	1	0	0	0

Patientennummer, Alter in Jahren zum Zeitpunkt der Therapie (*J.*), Geschlecht in weiblich (*w*) und männlich (*m*). Krankheitsdauer in Jahren (*J.*). Anzahl der fehlgeschlagenen Behandlungen, Krankheitsaktivität zu Beginn der Behandlung, dargestellt mit dem SLE-Krankheitsaktivitätsindex (*SLEDAI*). Als Wirksamkeitskriterien wurden angegeben: Serokonversion, Verschwinden von SLE-spezifischen Antikörpern (Anti-dsDNA [doppelsträngige Desoxyribonukleinsäure]), Definitionen der Remission bei systemischem Lupus erythematodes (DORIS-Remission [Definition of Remission in SLE]). Unerwünschte Ereignisse werden unter Zytokinfreisetzungssyndrom (*CRS*) und immunbedingtes Effektorzellneurotoxizitätssyndrom (*ICANS*) sowie Hämatotoxizitäten (Effektorzellhämatotoxizitätssyndrom [*ICAHT*]) in den Stufen 0 bis 4 aufgeführt. Anzahl an Infektion nach CD19-CAR-T-Zell-Therapie mit schweren Verläufen

*n.d.* not determined, *ZNS* Zentralnervensystem

### Wirksamkeit

Die Krankheitsaktivität bei SLE wurde anhand des Systemic Lupus Erythematosus Disease Activity Index 2000 (SLEDAI-2K; Bereich von 0 bis 105, wobei höhere Werte auf eine stärkere Krankheitsaktivität hinweisen), der Kriterien für einen Lupus Low Disease Activity State (LLDAS; SLEDAI ≤ 4 und tägliche Glukokortikoiddosierung < 5 mg) sowie der Remissionskriterien gemäß der Definition of Remission in SLE (DORIS; SLEDAI = 0, ärztliche Aktivitätsbeurteilung < 0,5 und tägliche Glukokortikoiddosierung < 5 mg) alle 3 Monate nach der CAR-T-Zell-Gabe beurteilt. Alle Organmanifestationen und serologischen Parameter wurden regelmäßig untersucht [19].

### Sicherheit

Die Einstufung des Zytokinfreisetzungssyndroms (CRS) und des Immuneffektorzell-assoziierten Neurotoxizitätssyndroms (ICANS) erfolgte nach den Konsenskriterien der American Society for Transplantation and Cellular Therapy (ASTCT) [20]. Die Knochenmarkstoxizität wurde nach den kürzlich definierten Kriterien der Immuneffektorzell-assoziierten Hämatotoxizität (ICAHT) eingestuft [21].

### Ethik

Die CAR-T-Zell-Behandlung wurde als „named patient use“ (AMG [Arzneimittelgesetz], § 21, Härtefallverordnung) durchgeführt. Die Erhebung und Auswertung der Daten unterliegen dem Ethikvotum der lokalen Ethikkommission der Universität Erlangen (334_18 B).

## Ergebnisse

Wir analysierten die ersten 11 SLE-PatientInnen (9 weiblich, 2 männlich, Altersspanne 18 bis 41 Jahre, demografische Daten in Tab. 1), die in unserem Zentrum mit CD19-CAR-T-Zellen (MB19,1) behandelt wurden. Die mediane Nachbeobachtungszeit betrug 2,5 Jahre (0,5 bis 4 Jahre). Zusammengefasst hatten die PatientInnen eine aktive Erkrankung mit einem medianen SLEDAI von 16 (8 bis 22), einem medianen Organbefall von 4 Organen (3 bis 6) sowie einem medianen Wert von 6 Vortherapien (4 bis 14). Es lagen Beteiligungen von Niere, Herz, Lunge, Gelenken, Haut, Muskeln, Knochenmark und/oder Zentralnervensystem (ZNS) vor. Die PatientInnen erhielten nach Lymphodepletion eine einmalige Infusion von CD19-CAR-T-Zellen, und alle erreichten innerhalb von 6 Monaten eine vollständige Remission gemäß DORIS-Kriterien. Unsere Langzeitbeobachtungen von bis zu 4 Jahren nach der Therapie zeigten einen Fall mit erneuter Krankheitsaktivität. Die anderen 10 PatientInnen verblieben in einer lang anhaltenden, medikamentenfreien Remission (SLEDAI-2K-Score: 0). Anti-dsDNA-Antikörper waren bei allen PatientInnen nicht mehr erhöht nachweisbar, teils vollständig serokonvertiert, die Komplementfaktor-C3-Werte normalisierten sich. Bei PatientInnen mit Lupusnephritis konnte ebenfalls eine deutliche Verbesserung der Nierenfunktion und Proteinurie, überwiegend eine komplette Rekonstitution, gesehen werden.

### Fallbericht der CD19-CAR-T-Zell-Therapie bei schwerer Multiorganbeteiligung des systemischen Lupus erythematodes (Abb. 1, Tab. 1 Pat. #8)

Die Zuweisung und stationäre Aufnahme der 35-jährigen Patientin erfolgte auf unsere rheumatologische Normalstation bei seit 2003 bekanntem systemischem Lupus erythematodes mit schwerem und refraktärem Verlauf. Vortherapien inkludierten Glukokortikoide, Hydroxychloroquin, Immunglobuline, Belimumab, Thalidomide, Methotrexat, Mycophenolat-Mofetil und Rituximab. Laborchemisch zeigten sich eine Proteinurie, Komplementverbrauch und Nachweis von dsDNA sowie Histonantikörpern. Es erfolgte ein umfassendes Organscreening inklusive Computertomographie (CT) des Thorax, Abdomensonographie, Echokardiographie, Bodyplethysmographie. Spirometrisch zeigten sich eine schwergradige obstruktive Ventilationsstörung sowie eine mittel- bis schwergradige Diffusionsstörung. Computertomographisch imponierten diffuse bipulmonale Milchglastrübungen.

**Abb. 1:**
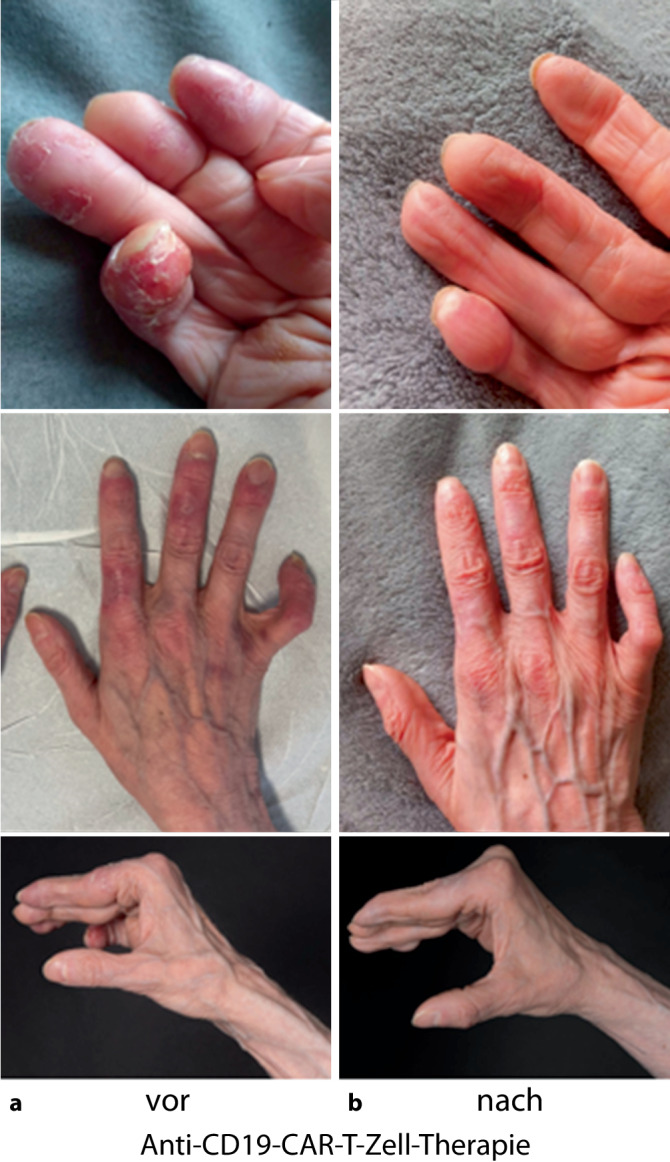
Vaskulitische und arthritische Aktivität, **a** vor CD19-CAR(chimärer Antigenrezeptor)-T-Zell-Therapie, **b** 6 Monate nach CD19-CAR-T-Zell-Therapie

Zusammenfassend manifestierten sich bei der Patientin trotz diverser Vortherapien und anhaltend hohen Steroidbedarfs eine schwere Vaskulitis mit mukokutanem Befall, Lawrence-Syndrom, Polyarthritis, Angioödem, Episkleritis, Bronchiolitis obliterans mit Langzeitsauerstoffbedarf und einer aktiven Lupusnephritis Klasse IV.

Bei refraktärem Verlauf und anhaltend hoher Krankheitsaktivität des SLE (SLEDAI-2K = 22) wurde bei fehlenden Therapieoptionen ein individueller Heilversuch mit CD19-CAR-T-Zellen vorgenommen. Es erfolgt eine lymphodepletierende Therapie mit Cyclophosphamid (1000 mg/m^2^ Tag−3) und Fludarabin (25 mg/m^2^ Tag −5, −4 und −3). Die Rückgabe der autologen CD19-CAR-T-Zellen erfolgt mit 1 × 10^6^ Zellen/kg Körpergewicht an Tag 1. Die CAR-T-Zellen expandierten und erreichten ihren Spitzenwert an Tag 10. Bereits im kurzfristigen Verlauf bemerkte die Patientin eine Verbesserung der vaskulitischen Läsionen an Haut und Schleimhäuten sowie gebesserte Motorik und ein Abklingen der arthritischen Beschwerden. Sie berichtete eine gebesserte Belastbarkeit mit abklingender Fatigue-Symptomatik (s. Abb. 1). In der Lungenfunktion zeigten sich eine Stabilisierung und letztlich Besserung. Die Langzeitsauerstofftherapie konnte 5 Monate nach der CAR-T-Zell-Therapie beendet werden. Laborchemisch normalisieren sich Entzündungsparameter, dsDNA und Komplementfaktoren. Die B‑Zellen waren vollständig depletiert und ab dem 225. Tag nach CAR-T-Zell-Infusion wieder im peripheren Blut nachweisbar. Die Patientin befand sich bei allen weiteren Kontrollen gemäß DORIS-Kriterien in therapiefreier Remission.

### Sicherheit

**Abb. 2:**
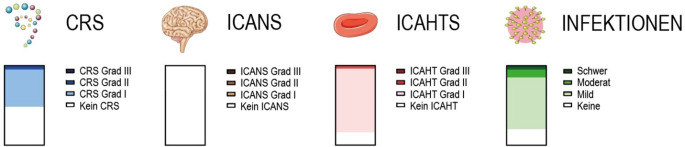
Nebenwirkung der CD19-CAR(chimärer Antigenrezeptor)-T-Zell-Therapie in illustrierter Häufigkeit bei 11 SLE(systemischer Lupus erythematodes)-PatientInnen. **a** „Cytokine release syndrome“ (*CRS*), eingeteilt in Schweregrade (kein CRS mit 45 %, Grad 1 mit 45 %, Grad 2 mit 10 %, Grad 3 mit 0 %), gefolgt von **b** „immune effector cell-associated neurotoxicity syndrome“ (*ICANS*), eingeteilt in Schweregrade (kein ICANS verzeichnet), gefolgt von **c** „immune effector cell-associated hematotoxicity syndrome“ (*ICAHTS*) als frühe Neutropenie (keine späten verzeichnet). Grad 1 bei 70 % sowie Grad 2 bei 10 % der PatientInnen, und gefolgt von **d** Infektionen, eingeteilt in keine, mild mit 65 %, moderat mit 10 % und schwer mit 10 % Häufigkeit bei den oben genannten PatientInnen

Trotz der vielversprechenden Wirksamkeit ist die CAR-T-Zell-Therapie mit potenziellen Sicherheitsrisiken verbunden, die eine sorgfältige Überwachung und ein erfahrenes Therapiemanagement erfordern. Die frühe Hämatotoxizität der CAR-T-Zell-Therapie wird vermutlich überwiegend durch eine kurze lymphozytotoxische Chemotherapie (bestehend aus Cyclophosphamid, Fludarabin) vom fünften bis dritten Tag vor der eigentlichen Infusion verursacht. Diese kann zu einer vorübergehenden Leukopenie, Neutropenie, Thrombozytopenie und Anämie führen. Dem folgen eine Expansion und Aktivierung der CAR-T-Zellen. Die zunächst von den aktivierten CAR-T-Zellen produzierten Zytokine aktivieren wiederum rasch andere Immunzellen, die daraufhin teils große Mengen an Effektorzytokinen und Chemokinen einschließlich Interleukin 6 ausschütten. Die daraus resultierende Zytokinfreisetzung ist in erster Linie für die sog. Immuneffektorzell(IEC)-assoziierten Nebenwirkungen verantwortlich, darunter das Zytokinfreisetzungssyndrom (CRS), das Neurotoxizitätssyndrom (ICANS) oder das hämophagozytische lymphohistiozytoseähnliche Syndrom (IEC-HS) [22, 23]. Dabei korreliert die Menge an verschiedenen Zytokinen mit der Hämatotoxizität (ICAHT) ([21]; Abb. 2). Nach der Infusion expandierten die CAR-T-Zellen bei unseren PatientInnen mit einem Spitzenwert um Tag 9 (Mittelwert) und nahmen danach rapide ab. Im langfristigen Verlauf waren die CAR-T-Zellen in sehr geringen Mengen, meist aber gar nicht mehr nachweisbar. Die mediane B‑Zell-Depletion dauerte 84 (83 bis 360) Tage. Von den 11 genannten SLE-PatientInnen hatten 5 (45 %) ein CRS ersten Grades, das durch die Gabe von Paracetamol oder Tocilizumab durchbrochen wurde. Ein CRS Grad 2 wurde nur 1‑malig beobachtet. Höhergradige CRS sind nicht aufgetreten. Bei unseren SLE PatientInnen konnten wir bisweilen kein ICANS feststellen. Initiale Neutropenien („early ICAHT“) traten bei 9 der 11 PatientInnen (82 %) auf, die sich spontan erholten. GCSF („granulocyte-colony stimulating factor“) wurde nicht benötigt. Aplastische Anämien, genauso wie Thrombopenien, fanden sich keine.

Eine B‑Zell-gerichtete Therapie birgt ebenso ein Risiko zur sekundären Hypogammaglobulinämie, die wir bei 6 (55 %) unserer PatientInnen beobachteten. Diese blieben meist nur leicht ausgeprägt und traten ohne eine vermehrte Infektanfälligkeit auf. Eine Immunglobulingabe war daher nur bei 3 (50 %) dieser genannten PatientInnen notwendig.

Die Komplikation mit der höchsten Sterblichkeit ist im Bereich der Lymphombehandlungen (abgesehen von einem Krankheitsrückfall oder Sekundärlymphom) auf Infektionen nach der CAR-T-Zell-Therapie zurückzuführen [24]. Wir verzeichneten im langjährigen Verlauf bisher überwiegend milde Infektionen. Am häufigsten handelte es sich um Infekte der oberen Atemwege oder Harnwegsinfektionen (7 PatientInnen, 64 %). Eine schwere Infektion mit stationärem Aufenthalt ließ sich 1‑malig auf eine Pneumonie (respiratorisches Synzytialvirus [RSV]) zurückführen. Insbesondere bei Autoimmunerkrankungen wie SLE müssen zudem mögliche Langzeitfolgen einer tiefgreifenden B‑Zell-Depletion berücksichtigt werden. Da die Zielstruktur von anti-CD19-Reifestadien der B‑Zelle von der Vorläufer-B-Zelle bis zu Plasmablasten umfasst, bleiben langlebige, CD19-negative Plasmazellen verschont. Dies kann erklären, warum wir bis dato stabile und protektive Impftiter, beispielsweise für Pneumokokken, Mumps, Masern, Röteln oder COVID, nachweisen konnten.

## Diskussion

In den vergangenen 4 Jahren hat die Behandlung mit CD19-CAR-T-Zellen eine bislang beispiellose Wirksamkeit bei PatientInnen mit schwer verlaufenden B‑Zell-getriebenen Autoimmunkrankheiten gezeigt. Bei den oben genannten 11 PatientInnen mit SLE wurde ein vollständiges Abklingen der Krankheitssymptome innerhalb der ersten 6 Monate nach Infusion beobachtet. Bemerkenswert ist, dass alle PatientInnen ihre immunsuppressive Medikation erfolgreich absetzen konnten. Lediglich bei einer Patientin trat ein Rezidiv der Krankheitsaktivität auf. Neben schwerwiegenden Organmanifestationen wie der Lupusnephritis, die unter der CAR-T-Zell-Therapie bislang erfolgreich kontrolliert werden konnte, wurde auch ein erfolgreicher Therapieversuch bei einer zentralnervösen Manifestation des SLE beschrieben. Ein Patient mit transverser Myelitis und progredienter Tetraplegie über einen Zeitraum von 3 Monaten zeigte keine Besserung unter herkömmlichen Therapien (Hochdosis-Glukokortikoide, Rituximab, Cyclophosphamid, Plasmapherese). Im Rahmen eines individuellen Heilversuchs erhielt er eine CD19-CAR-T-Zell-Therapie. Bereits 4 Wochen nach der Infusion konnte der Patient wieder selbstständig sitzen, und 7 Wochen danach war das Gehen mit Hilfsmitteln möglich [25]. Dieser Fall eröffnet neue Perspektiven für die Behandlung lebensbedrohlicher ZNS(Zentralnervensystem)-Beteiligungen bei SLE. Auch der erstmalige Einsatz der Therapie bei einer pädiatrischen Patientin mit SLE verlief erfolgreich. Bei der 15-jährigen Patientin zeigte sich ein vergleichbares Sicherheits- und Wirkungsprofil wie bei Erwachsenen [26].

In einer multizentrischen Phase-I-Studie wurden 13 PatientInnen mit SLE und aktiver Lupusnephritis mit Anti-BCMA(B-Zell-Reifungsantigen)-CD19-compound-CAR-T-Zellen behandelt. Bei 12 von 13 PatientInnen konnte eine symptom- und medikamentenfreie Remission erreicht werden [13]. Unsere hier genannte Fallserie und Langzeitbeobachtungen von bis zu 4 Jahren veranschaulichen die grundsätzliche Möglichkeit, SLE durch eine einzige Infusion mit CD19-CAR-T-Zellen zu behandeln und eine tiefgreifende Depletion von B‑Zellen zu bewirken. Darüber hinaus ist induzierte B‑Zell-Depletion bei SLE im Gegensatz zum Einsatz bei Lymphomen nur von kurzer Dauer, und bisher ist es trotz Rekonstitution der B‑Zellen in 10 von diesen 11 Fällen (91 %) zu keinem Wiederauftreten der Krankheit gekommen. Die erzielte, stabile Remission wird durch einen anhaltenden Rückgang pathogener Autoantikörper unterstützt. Zusätzlich sprechen das Auftreten eines naiven, nicht klassenwechselnden B‑Zell-Repertoires, das Verschwinden zirkulierender Plasmablasten sowie die Herunterregulierung krankheitsspezifischer schwerer und leichter Immunglobulinketten für einen immunologischen Neustart [10, 27]. Obwohl die Therapie sowohl pathogene als auch nichtpathogene B‑Zellen eliminiert, erfolgt die Regeneration des B‑Zell-Systems offenbar in Abwesenheit autoreaktiver Zellklone.

Trotz der Wirksamkeit sind Bedenken hinsichtlich der Toxizität bei AutoimmunpatientInnen von entscheidender Bedeutung, da die unmittelbare Bedrohung durch die Krankheit anders gewertet werden sollte als bei Malignomen. Trotz ähnlicher Pharmakokinetik der Lymphodepletion und der CD19-CAR-T-Zellen deuten vorläufige Daten auf ein günstigeres kurz- und mittelfristiges Nebenwirkungsprofil der CAR-T-Zell-Therapie bei SLE hin, einschließlich einer schnelleren Erholung des adaptiven Immunsystems. SLE-PatientInnen wiesen eine geringere Inzidenz höhergradiger Nebenwirkungen auf [28].

Eine möglicherweise bisher nicht beschriebene neue Form der Toxizität, „local immune effector cell-associated toxicity syndrome“ (LICATS), trat ausschließlich während der B‑Zell-Aplasie-Phase auf und betraf nur Organe, die zuvor von der jeweiligen Grunderkrankung betroffen waren. Die am häufigsten betroffenen Organe bei SLE-PatientInnen waren die Haut und die Nieren. Die meisten LICATS waren leicht. Alle LICATS bildeten sich ohne Folgeerscheinungen vollständig zurück [29].

Das Auftreten von Infektionen bleibt die potenziell schwerste Langzeittoxizität einer CAR-T-Zell-Therapie [24]. Infektionen sind unter anderem mit einer anhaltenden Neutropenie, einer schlechten Rekonstitution von T‑Zellen und einer anhaltenden Reduktion oder gar einer anhaltenden Depletion von B‑Zellen nach einer CD19-CAR-T-Zell-Therapie verbunden [30]. Ein erhöhtes Infektionsrisiko kann auch auf das Lymphozytendepletionsschema und eine daraus resultierende Hypogammaglobulinämie zurückgeführt werden [31, 32]. Wir dokumentierten bei den genannten PatientInnen eine schwere Infektionen aufgrund einer RSV-Pneumonie, die im Rahmen eines stationären Aufenthaltes adäquat versorgt wurde und folgenlos ausheilte. Bei allen SLE-PatientInnen trat eine B‑Zell-Rückkehr im Median nach 150 Tagen auf.

Weitere Daten aus klinisch kontrollierten Studien bleiben abzuwarten. Die erste Phase-I/II-Studie der westlichen Welt, CASTLE-Studie (Eduract 2022-001366-35), mit autologer CD19-CAR-T-Zell-Therapie wird hierzu weitere wichtige Erkenntnisse bringen. Das Therapiekonzept wurde ebenso erfolgreich bei weiteren Erkrankungen, auch neurologischen Erkrankungen wie Myasthenia gravis eingesetzt [33]. Neben dem vielversprechenden Wirksamkeitsprofil und akzeptablen Sicherheitsprofil kann die Behandlung mit CD19-CAR-T-Zellen die physische und psychische Gesundheit von SLE-PatientInnen verbessern und bei erzielter medikamentenfreier Remission eine Reduktion der sozioökonomischen Belastung bedeuten. In einer kürzlich veröffentlichten Studie berichteten Wang et al. [34] über den ersten Einsatz allogener CD19-CAR-T-Zellen bei Patienten mit therapieresistenten Autoimmunerkrankungen und wiesen erneut deren Wirksamkeit bei guter Verträglichkeit nach. Diese Ergebnisse deuten darauf hin, dass allogene CAR-T-Zellen eine skalierbare Behandlungsoption für Autoimmunerkrankungen darstellen könnten [35]. Einsatz finden CD19-CAR-T-Zellen mittlerweile auch bei weiteren Autoimmunerkrankungen wie systemischer Sklerose, idiopathischer inflammatorischer Myositis, insbesondere im Rahmen von kontrollierten klinischen Studien auch an weiteren universitären Zentren. Nicht zuletzt bleibt zu erwähnen, dass der Einsatz von CD19-CAR-T-Zellen als Möglichkeit der B‑Zell-Depletion bei schweren refraktären Fällen des SLE in der aktuellen S3-Leitlinie der DGRh [34].

## Fazit für die Praxis

- Der Einsatz von CD19-CAR(chimärer Antigenrezeptor)-T-Zellen bei systemischem Lupus erythematodes (SLE) mit schwerem und therapierefraktärem Verlauf ist wirksam und sicher. Diese Empfehlung findet sich auch in der aktuellen S3-Leitlinie Management des SLE der DGRh (Deutsche Gesellschaft für Rheumatologie und Klinische Immunologie e. V.).
- Bei allen 11 PatientInnen mit SLE wurde innerhalb von 6 Monaten ein vollständiges Abklingen der Krankheitssymptome beobachtet. Bemerkenswert ist, dass 10 von 11 PatientInnen ihre immunsuppressive Medikation erfolgreich absetzen konnten, ohne dass es zu Rückfällen oder einer Verschlechterung der Erkrankung kam.
- Obwohl diese Daten neue Erkenntnisse zur kurz- und langfristigen Sicherheit und Wirksamkeit der CD19-CAR-T-Zell-Therapie bei SLE liefern, sind kontrollierte klinische Studien erforderlich.

## Data Availability

Die erhobenen Datensätze können auf begründete Anfrage in anonymisierter Form beim korrespondierenden Autor angefordert werden.
